# Skin autofluorescence predicts incident type 2 diabetes, cardiovascular disease and mortality in the general population

**DOI:** 10.1007/s00125-018-4769-x

**Published:** 2018-11-21

**Authors:** Robert P. van Waateringe, Bernardina T. Fokkens, Sandra N. Slagter, Melanie M. van der Klauw, Jana V. van Vliet-Ostaptchouk, Reindert Graaff, Andrew D. Paterson, Andries J. Smit, Helen L. Lutgers, Bruce H. R. Wolffenbuttel

**Affiliations:** 1 Department of Endocrinology, University of Groningen, University Medical Center Groningen, Hanzeplein 1, P.O. Box 30001, HPC AA31 9700 RB Groningen, the Netherlands; 2 Department of Internal Medicine, University of Groningen, University Medical Center Groningen, Groningen, the Netherlands; 3 Program in Genetics and Genome Biology, Hospital for Sick Children, Toronto, ON Canada; 4 Department of Internal Medicine, Medical Center Leeuwarden, Leeuwarden, the Netherlands

**Keywords:** Ageing, Cardiovascular, Diabetes, Mortality, Prediction, Skin autofluorescence

## Abstract

**Aims/hypothesis:**

Earlier studies have shown that skin autofluorescence measured with an AGE reader estimates the accumulation of AGEs in the skin, which increases with ageing and is associated with the metabolic syndrome and type 2 diabetes. In the present study, we examined whether the measurement of skin autofluorescence can predict 4 year risk of incident type 2 diabetes, cardiovascular disease (CVD) and mortality in the general population.

**Methods:**

For this prospective analysis, we included 72,880 participants of the Dutch Lifelines Cohort Study, who underwent baseline investigations between 2007 and 2013, had validated baseline skin autofluorescence values available and were not known to have diabetes or CVD. Individuals were diagnosed with incident type 2 diabetes by self-report or by a fasting blood glucose ≥7.0 mmol/l or HbA_1c_ ≥48 mmol/mol (≥6.5%) at follow-up. Participants were diagnosed as having incident CVD (myocardial infarction, coronary interventions, cerebrovascular accident, transient ischaemic attack, intermittent claudication or vascular surgery) by self-report. Mortality was ascertained using the Municipal Personal Records Database.

**Results:**

After a median follow-up of 4 years (range 0.5–10 years), 1056 participants (1.4%) had developed type 2 diabetes, 1258 individuals (1.7%) were diagnosed with CVD, while 928 (1.3%) had died. Baseline skin autofluorescence was elevated in participants with incident type 2 diabetes and/or CVD and in those who had died (all *p* < 0.001), compared with individuals who survived and remained free of the two diseases. Skin autofluorescence predicted the development of type 2 diabetes, CVD and mortality, independent of several traditional risk factors, such as the metabolic syndrome, glucose and HbA_1c_.

**Conclusions/interpretation:**

The non-invasive skin autofluorescence measurement is of clinical value for screening for future risk of type 2 diabetes, CVD and mortality, independent of glycaemic measures and the metabolic syndrome.

**Electronic supplementary material:**

The online version of this article (10.1007/s00125-018-4769-x) contains peer-reviewed but unedited supplementary material, which is available to authorised users.

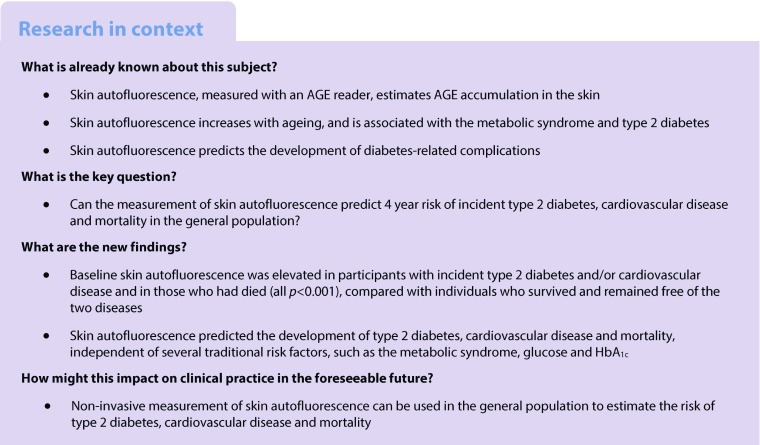



## Introduction

The worldwide prevalence of type 2 diabetes is increasing rapidly; it is predicted to be close to 650 million in 2040. Cardiovascular complications are the main drivers of increased morbidity and premature mortality in diabetes [1–3]. Several risk factors, such as degree of obesity, fasting blood glucose level and presence of the metabolic syndrome, predict the development of type 2 diabetes and cardiovascular disease (CVD), and several risk scores have been developed to increase the reliability of disease prediction [4–7].

In the last two decades, the role of AGEs in ageing and the pathophysiology of diabetes-related complications has been studied extensively. AGEs are formed in a multistep process by glycation and oxidation of free amino groups of proteins, lipids and nucleic acids. In addition to the classic Maillard reaction, AGEs are formed through the reaction of amino groups with α-dicarbonyls, such as 3-deoxyglucosone, methylglyoxal and glyoxal [8–10]. AGEs may form cross-links between tissue proteins in the vascular wall, causing increased vascular stiffness and elevated BP [11, 12]. Moreover, binding of circulating AGE to its receptor (receptor for AGE [RAGE]) and uptake into the vessel wall may accelerate the progression of atherosclerosis [13, 14]. AGEs may also induce beta cell damage by increasing inflammation and oxidative stress and thereby contribute to worsening of hyperglycaemia [15, 16].

The accumulation of AGEs can be assessed non-invasively by measuring skin autofluorescence (SAF) [17]. This method is based on the fluorescent properties of certain AGEs accumulated in dermal tissue. Validation studies have shown that SAF is strongly related to AGE levels in skin biopsies [18]. SAF increases with ageing, and is elevated in people with type 2 diabetes compared with age-matched control individuals [19, 20]. We have recently demonstrated that SAF is already elevated in people without diabetes but with the metabolic syndrome and is associated with its individual components [21]. SAF is strongly associated with long-term cardiovascular complications and mortality in type 2 diabetes [19, 22–24].

Long-term prospective studies on the value of SAF to predict development of type 2 diabetes, CVD and mortality in the general population are lacking. However, SAF was associated with increased mortality and cardiovascular events in specific groups and, for example, predicted amputations in individuals with peripheral artery disease [25, 26].

Because of the promise of SAF as a valuable biomarker, the goal of this study was to assess whether SAF was able to predict the development of type 2 diabetes, CVD and mortality in the general population. For this, we performed an extensive prospective follow-up study of individuals participating in the Dutch Lifelines Cohort Study.

## Methods

### Participants

Participants from the Lifelines Cohort Study, a large population-based study in the northern region of the Netherlands (electronic supplementary material [ESM] Methods), were included [27]. At baseline, both physical examination and extensive questionnaire data were collected [28]. All individuals provided written informed consent before participating in the study, which was approved by the Medical Ethics Review Committee of the University Medical Center Groningen.

For the present study, we evaluated 82,904 participants of Western European descent between 18 and 90 years of age, who underwent baseline investigations between 2007 and 2013, and for whom validated SAF measurement was available at baseline and prospective follow-up was performed between January 2014 and January 2018. There were no relevant differences in sex distribution, age and glycaemic variables between those with and without SAF measurements. We excluded participants who, at baseline, had clinical CVD (*n* = 1861) and/or type 1 diabetes (*n* = 177) and/or type 2 diabetes (*n* = 2557) or reported a history of gestational diabetes (*n* = 134) or MODY (*n* = 4). Furthermore, individuals with new type 1 diabetes (*n* = 12) and a new history of gestational diabetes (*n* = 55) at follow-up were excluded, as well as those without documented follow-up (*n* = 5530). This resulted in 72,880 individuals available for analyses (ESM Fig. 1). Of these, 59,583 participated in the second screening, filled in the follow-up questionnaires and underwent follow-up examination with detailed BP measurement and laboratory examinations (ESM Methods), while only interim questionnaire data were available for 13,297 individuals. Median follow-up was 4 years, with a range of 0.5–10 years, for a total of 274,629 participant-years (ESM Fig. 2). Median follow-up for the 59,583 participants who had completed the second screening visit and measurements was 4.1 years. Follow-up measurements of fasting blood glucose and HbA_1c_ were available for 55,759 (76.5%) and 56,086 (77%) participants, respectively.

### Clinical examination

At both baseline and follow-up examination, participants completed a self-administered questionnaire on medical history, past and current diseases, and health behaviour. Medication use was verified at baseline by a certified research assistant and scored using the Anatomical Therapeutic Chemical (ATC) Classification System. Information regarding smoking behaviour (never, former and current smoking) and quantity smoked, as well as coffee consumption (cups/day), was collected from the questionnaires [29]. Weight was measured to the nearest 0.1 kg and height and waist circumference to the nearest 0.5 cm, with participants wearing light clothing and no shoes. BMI was calculated as kg/m^2^. Systolic and diastolic BP and heart rate were measured every minute for 10 min in the supine position using an automated Dinamap monitor (GE Healthcare, Freiburg, Germany). The average of the last three readings was recorded for each BP variable and heart rate.

### Skin autofluorescence

SAF was measured non-invasively using an AGE reader (Diagnoptics Technologies, Groningen, the Netherlands), as described previously [17, 20]. The AGE reader illuminates a skin surface of approximately 4 cm^2^, guarded against surrounding light, with an excitation light source with wavelength between 300 and 420 nm (peak intensity at ~370 nm). Emission light and reflected excitation light from the skin are measured with an internal spectrometer in the range 300–600 nm. SAF was based on the ratio of the average emitted light intensity per nm in the range 420–600 nm and the average reflected light intensity per nm in the range 300–420 nm, multiplied by 100, and is expressed in arbitrary units (AU), taking skin colour into account [30]. Previous studies have shown an error rate of 5% when repeated SAF measurements were taken over a single day in control participants and individuals with diabetes [17]. More details about the number of machines and validation of measurements are given in the ESM Methods. Age-adjusted SAF levels (*z* scores) were calculated separately for men and women, based on the total population.

### Biochemical measurements

Blood samples were taken in the fasting state between 08:00 and 10:00 hours and transported to the laboratory facility at room temperature or 4°C, depending on the sample requirements. On the same day, HbA_1c_ (EDTA-anticoagulated) was analysed using an NGSP-certified turbidimetric inhibition immunoassay on a Cobas Integra 800 CTS analyser (Roche Diagnostics Nederland, Almere, the Netherlands). Serum creatinine was measured on a Roche Modular P chemistry analyser (Roche, Basel, Switzerland) and renal function was calculated as estimated (e)GFR with the formula developed by the Chronic Kidney Disease Epidemiology Collaboration (CKD-EPI) [31]. Total cholesterol and HDL-cholesterol were measured using an enzymatic colorimetric method, triacylglycerol using a colorimetric UV method, and LDL-cholesterol using an enzymatic method, on a Roche Modular P chemistry analyser (Roche). Fasting blood glucose was measured using a hexokinase method.

### Calculations, definitions and statistical analyses

Diagnosis of the metabolic syndrome was established if a participant at baseline satisfied at least three out of five criteria according to the modified guidelines of the National Cholesterol Education Programs Adults Treatment Panel III (NCEP ATPIII criteria): (1) systolic BP ≥130 mmHg and/or diastolic BP ≥85 mmHg and/or use of antihypertensive medication; (2) HDL-cholesterol levels <1.03 mmol/l in men and <1.30 mmol/l in women and/or use of lipid-lowering medication influencing HDL-cholesterol levels; (3) triacylglycerol levels ≥1.70 mmol/l and/or use of triacylglycerol-lowering medication; (4) waist circumference ≥102 cm in men and ≥88 cm in women; (5) fasting glucose level ≥5.6 mmol/l and/or use of blood glucose-lowering medication and/or diagnosis of type 2 diabetes [32]. Incident type 2 diabetes was based on either self-report or fasting blood glucose ≥7.0 mmol/l and/or HbA_1c_ ≥48 mmol/mol (≥6.5%) at follow-up evaluation. Incident CVD was defined as present when participants reported myocardial infarction, percutaneous transluminal coronary angioplasty (PTCA), stent positioning, coronary artery bypass grafting (CABG), transient ischaemic attack (TIA), cerebrovascular accident (CVA), intermittent claudication or peripheral artery vascular surgery. Vital status was ascertained with the Municipal Personal Records Database (GBA). Data on cause of death were not available. The incidence of type 2 diabetes, CVD and mortality was calculated separately and as a composite outcome for all age-decade groups (18–29, 30–39, 40–49, 50–59, 60–69, 70–79, ≥80 years)..

All analyses were conducted using PASW Statistics (Version 22, IBM, Armonk, NY, USA). Data are presented as mean ± SD, or median and interquartile range (IQR) when not normally distributed. Means were compared between groups with ANOVA. When variables were not normally distributed, medians were compared using the non-parametric Mann–Whitney *U* test. The *χ*^2^ test was used to analyse categorical variables. Uni- and multivariate logistic regression analyses were performed to examine the association between SAF and the composite outcome of incident type 2 diabetes, CVD and mortality, as well as these outcomes separately, while adjusting for relevant clinical, biochemical and lifestyle risk factors. In our models, we adjusted for the most important determinants of SAF, i.e. age (model 1, used as a basic model), additionally adjusted for presence of the metabolic syndrome (model 2), glycaemic variables (model 3a/b), confounding non-biochemical factors (model 4) and all relevant factors (model 5). As age is an important factor influencing not only SAF measurements, but also the absolute incidence of events, we calculated the association between SAF and outcome according to four clinically relevant age groups, considered as low, intermediate, high and very high risk: ≤35 years; 36–50 years; ≥51 years; and ≥61 years. Values of *p* < 0.05 were considered statistically significant.

## Results

### Incidence of type 2 diabetes, CVD and mortality

The incidence of all outcomes is shown in Table 1. An individual may have had more than one outcome. After a median follow-up of 4 years, 1056 individuals had developed type 2 diabetes (1.4%). Of those, 525 reported that they had been diagnosed with type 2 diabetes between the baseline visit and the follow-up measurement, while in those not reporting a diagnosis of diabetes, fasting blood glucose ≥7.0 mmol/l was observed in 408 participants, elevated HbA_1c_ ≥48 mmol/mol (≥6.5%) in 268, and either elevated blood glucose or HbA_1c_ in 531 participants.

**Table 1:** Clinical endpoints as defined in the study

Clinical endpoint	*n*
No type 2 diabetes, CVD or death	69,749
Incident type 2 diabetes only	977
Incident CVD only	1171
Death^a^	874
Incident type 2 diabetes and CVD	55
Type 2 diabetes and death	22
CVD and death	30
Type 2 diabetes, CVD and death	2

72,880 participants in total

^a^Without/before ascertainment of diabetes or CVD status

**Fig. 1:**
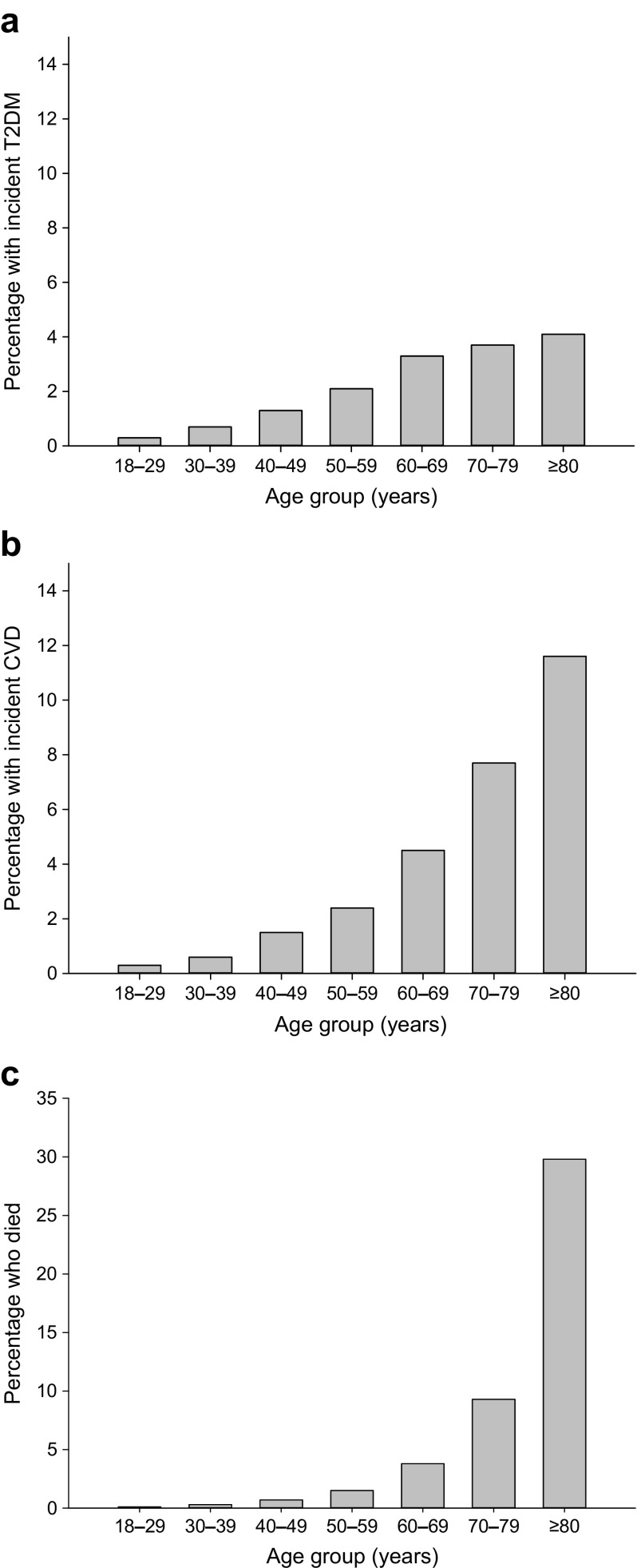
Proportion of participants per age decade (**a**) with incident type 2 diabetes, (**b**) with incident CVD and (**c**) who died. T2DM, type 2 diabetes

**Fig. 2:**
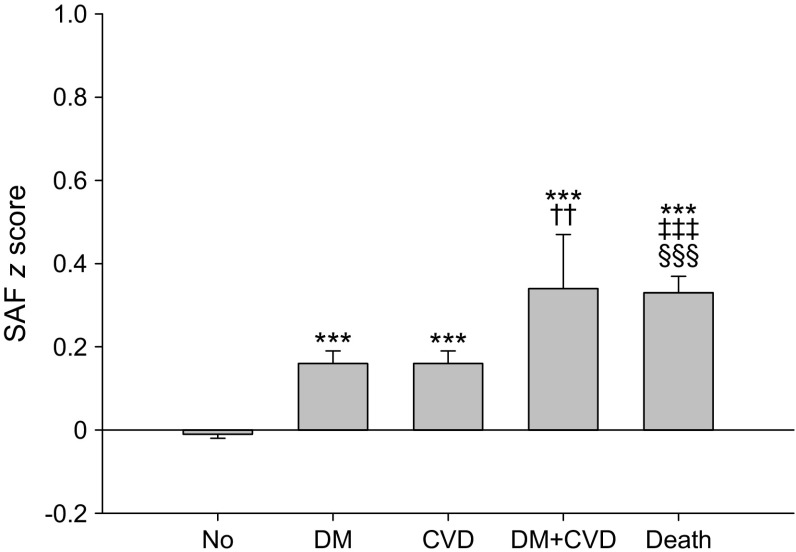
Baseline SAF *z* scores according to diabetes, CVD and vital status at 4 years of follow-up. Data are presented as means ± standard error. No type 2 diabetes/CVD (No), *n* = 69,749; type 2 diabetes (DM), *n* = 977; CVD, *n* = 1171; DM + CVD, *n* = 55; death, *n* = 928. ***p < 0.001 vs no type 2 diabetes/CVD group; ^††^*p* < 0.005 (women only) vs DM group; ^‡‡‡^*p* < 0.001 vs DM group; ^§§§^*p* < 0.001 vs CVD group

Individuals with incident type 2 diabetes were significantly older at baseline than participants who did not develop type 2 diabetes (51.8 ± 11.4 years vs 43.7 ± 12.0 years, *p* < 0.001), and had a higher baseline BMI, fasting glucose and HbA_1c_ (all *p* < 0.001, Table 2). Moreover, the prevalence of the metabolic syndrome was also higher. As expected, the incidence of type 2 diabetes increased with age, and was between 3.3% and 4.1% in the three highest age decades (Fig. 1a). Mean baseline SAF *z* score was 0.16 ± 0.95 in participants with incident type 2 diabetes and −0.01 ± 0.81 in individuals who remained healthy (*p* < 0.001, Fig. 2).

**Table 2:** Clinical characteristics of the study population at baseline in relation to outcome status

Characteristic	Incident			
	None	T2D	CVD	Death
Sex (*n*; male/female)	28,021/41728	524/532	637/621	489/439
Men (%)	40.2	49.6	50.6	52.7
Age (years)	43.7 ± 12.0	51.8 ± 11.4	54.2 ± 12.0	58.0 ± 12.7
BMI (kg/m^2^)	25.8 ± 4.1	29.7 ± 5.0	27.1 ± 4.0	26.8 ± 4.3
Waist (cm)	90 ± 12	101 ± 13	95 ± 12	95 ± 13
Systolic BP (mmHg)	125 ± 15	134 ± 16	133 ± 17	134 ± 18
Diastolic BP (mmHg)	74 ± 9	78 ± 10	77 ± 10	77 ± 10
Heart rate (bpm)	71 ± 11	73 ± 11	71 ± 11	71 ± 12
Creatinine (μmol/l)	73 ± 13	75 ± 15	76 ± 14	77 ± 22
eGFR (ml/min)	97 ± 15	92 ± 15	90 ± 15	87 ± 16
Total cholesterol (mmol/l)	5.1 ± 1.0	5.3 ± 1.0	5.4 ± 1.0	5.4 ± 1.0
HDL-cholesterol (mmol/l)	1.49 ± 0.39	1.29 ± 0.37	1.43 ± 0.40	1.46 ± 0.41
LDL-cholesterol (mmol/l)	3.2 ± 0.9	3.4 ± 0.9	3.6 ± 1.0	3.5 ± 0.9
Triacylglycerol (mmol/l)	0.96 (0.70–1.36)	1.41 (1.02–2.00)	1.16 (0.84–1.63)	1.12 (0.84–1.62)
Glucose (mmol/l)	4.9 ± 0.5	5.7 ± 0.7	5.1 ± 0.5	5.1 ± 0.5
HbA_1c_ (mmol/mol)	36 ± 3	40 ± 4	38 ± 3	38 ± 4
HbA_1c_ (%)	5.5 ± 0.3	5.9 ± 0.3	5.7 ± 0.3	5.7 ± 0.3
Current smokers (%)	20.4	22.3	25.8	26.9
Former smokers (%)	30.8	39.4	40.6	40.4
Metabolic syndrome (%)	12.8	57.5	25.1	25.6
Skin autofluorescence (AU)	1.90 ± 0.42	2.13 ± 0.45	2.18 ± 0.47	2.33 ± 0.52
SAF *z* score	−0.01 ± 0.81	0.16 ± 0.95	0.16 ± 0.96	0.33 ± 1.13

Data are presented as mean ± SD, median (IQR), number or %

*p* < 0.001 vs the group without incident type 2 diabetes, CVD or death in all analyses (except heart rate) by ANOVA

T2DM, type 2 diabetes

In the same population, 1258 individuals (1.7%) had developed CVD at follow-up (Table 1). Participants with incident CVD were significantly older at baseline, had a higher waist circumference, higher systolic BP and diastolic BP, higher lipid levels and a lower eGFR (*p* < 0.001, Table 2). As expected, incidence of CVD increased with age, and was between 4.5% and 11.6% in the three highest age decades (Fig. 1b). Mean baseline SAF *z* score was 0.16 ± 0.96 among the population with incident CVD vs −0.01 ± 0.81 in participants who did not develop CVD or type 2 diabetes (*p* < 0.001, Fig. 2). In total, 55 individuals developed both type 2 diabetes and CVD, and these had the highest baseline SAF *z* scores (*p* < 0.001 vs no disease, *p* = 0.004 vs type 2 diabetes [in women only], Fig. 2).

Death was reported in 928 individuals (1.3%). As expected, mortality increased with age (Fig. 1c). Participants who died were older at baseline, had higher BP, were more likely to have impaired renal function/low eGFR and were more frequently current smokers. They also had higher SAF levels, even when corrected for age, than individuals who developed type 2 diabetes or CVD or remained without these disorders (Table 2).

ESM Table 1 details the mean age and SAF levels according to each age group. In almost all age groups, SAF was significantly higher (*p* < 0.0001) in those participants who developed an event (the composite outcome of incident type 2 diabetes, CVD and mortality) compared with those who remained free from these events.

### Association and prediction

Table 3 shows the results of the univariate and multivariate associations between SAF and clinical, biochemical and lifestyle factors and the composite outcome of incident type 2 diabetes, CVD and mortality. Univariate analyses showed that SAF was strongly associated with these outcomes (OR 3.84, 95% CI 3.57, 4.11, *p* = 1.5 × 10^−307^). This association remained significant after adjusting for age (model 1), age and the metabolic syndrome (model 2) and also after adjusting for age, fasting glucose (OR 1.79, 95% CI 1.64, 1.96, *p* = 1.1 × 10^−37^) or HbA_1c_ (OR 1.78, 95% CI 1.63, 1.95, *p* = 6.6 × 10^−38^). Additional regression models revealed that the association also remained significant when adjusted for sex, waist circumference and current smoking (model 4), as well as all for other variables including systolic BP, plasma lipids, eGFR and coffee consumption (model 5, OR 1.54, 95% CI 1.40, 1.70, *p* = 3.9 × 10^−18^). In the composite multivariate model 5, age, glucose, waist circumference, current smoking, systolic blood pressure and triacylglycerol were most strongly associated with the composite outcome (Table 3).

**Table 3:** Univariate and multivariate logistic regression analyses for the composite primary outcome (incident type 2 diabetes, CVD or death) at a median of 4 years’ follow-up

Analysis	*n*	OR	95% CI	*p* value
Univariate				
SAF (AU)	72,880	3.84	3.57, 4.11	1.5 × 10^−307^
Age (years)	72,880	1.07	1.07, 1.08	<1.0 × 10^−350^
Male sex (y/n)	72,880	1.53	1.42, 1.64	3.9 × 10^−31^
BMI (kg/m^2^)	72,866	1.10	1.09, 1.10	2.3 × 10^−141^
Waist circumference (cm)	72,866	1.05	1.04, 1.05	3.0 × 10^−227^
Glucose (mmol/l)	72,223	4.02	3.77, 4.29	<1.0 × 10^−350^
HbA_1c_ (mmol/mol)	72,254	1.26	1.24, 1.27	<1.0 × 10^−350^
SBP (mmHg)	72,853	1.03	1.03, 1.04	1.0 × 10^−199^
DBP (mmHg)	72,853	1.04	1.04, 1.04	1.5 × 10^−100^
Heart rate (b/min)	72,853	1.00	1.00, 1.01	0.021
Cholesterol (mmol/l)	72,446	1.33	1.29, 1.38	5.8 × 10^−60^
Triacylglycerol (mmol/l)	72,446	1.35	1.31, 1.40	5.2 × 10^−81^
eGFR (ml/min)	72,423	0.97	0.97, 0.97	1.0 × 10^−155^
Former smoker (y/n)	72,127	1.51	1.40, 1.62	9.0 × 10^−28^
Current smoker (y/n)	72,127	1.29	1.19, 1.40	2.4 × 10^−9^
Coffee consumption (cups/day)	71,396	1.09	1.07, 1.11	1.3 × 10^−27^
Metabolic syndrome (y/n)	72,405	3.76	3.48, 4.06	1.5 × 10^−250^
Multivariate model 1^a^	72,880			
SAF (AU)		1.88	1.72, 2.05	4.4 × 10^−46^
Age (years)		1.06	1.06, 1.06	1.4 × 10^−241^
Multivariate model 2^b^	72,405			
SAF (AU)		1.77	1.62, 1.93	1.2 × 10^−36^
Age (years)		1.06	1.05, 1.06	1.8 × 10^−209^
Metabolic syndrome (y/n)		2.80	2.58, 3.03	3.1 × 10^−142^
Multivariate model 3a^c^	72,223			
SAF (AU)		1.79	1.64, 1.96	1.1 × 10^−37^
Age (years)		1.05	1.05, 1.05	2.0 × 10^−160^
Glucose (mmol/l)		2.90	2.71, 3.11	1.7 × 10^−210^
Multivariate model 3b^c^	72,254			
SAF (AU)		1.78	1.63, 1.95	6.6 × 10^−38^
Age (years)		1.05	1.04, 1.05	8.3 × 10^−128^
HbA_1c_ (mmol/mol)		1.16	1.14, 1.17	1.3 × 10^−126^
Multivariate model 4^d^	72,113			
SAF (AU)		1.55	1.41, 1.69	9.7 × 10^−21^
Age (years)		1.06	1.06, 1.07	7.7 × 10^−229^
Male sex (y/n)		1.10	1.02, 1.19	0.013
Waist circumference (cm)		1.04	1.03, 1.04	2.5 × 10^−104^
Current smoker (y/n)		1.62	1.48, 1.78	3.6 × 10^−26^
Multivariate model 5^e^	70,612			
SAF (AU)		1.54	1.40, 1.70	3.9 × 10^−18^
Age (years)		1.06	1.05, 1.06	1.4 × 10^−107^
Glucose (mmol/l)		2.37	2.20, 2.55	1.3 × 10^−112^
Current smoker (y/n)		1.61	1.46, 1.77	8.3 × 10^−23^
Waist circumference (cm)		1.02	1.02, 1.02	2.6 × 10^−26^
Male sex (y/n)		0.93	0.86, 1.02	0.108
SBP (mmHg)		1.01	1.01, 1.01	3.6 × 10^−10^
Cholesterol (mmol/l)		0.93	0.89, 0.97	0.001
Triacylglycerol (mmol/l)		1.15	1.10, 1.19	5.4 × 10^−13^
eGFR (ml/min)		1.00	1.00, 1.01	0.176
Coffee consumption (cups/day)		0.99	0.97, 1.00	0.135

Baseline risk factors were used to predict the median 4 year risk of the composite outcome of type 2 diabetes, CVD and death

SAF, age, glucose, HbA_1c_, waist circumference, systolic BP, cholesterol, triacylglycerol, eGFR and coffee consumption (cups/day) were defined as continuous variables. Male sex, current smoker (vs never smoker) and the metabolic syndrome were defined as categorical variables

^a^Age-corrected

^b^Including the metabolic syndrome

^c^Including glycaemic measures

^d^Without biochemical markers

^e^With all variables

DBP, diastolic BP; SBP, systolic BP; y/n, yes/no

Additionally, we assessed the relationship between SAF and the three individual outcomes separately (Table 4). In a univariate model, SAF was most strongly associated with death (OR 5.10, 95% CI 4.56, 5.70, *p* = 4.1 × 10^−181^). This association remained highly significant after adjusting for age (model 1), presence of the metabolic syndrome (model 2), glycaemic variables (model 3a/b) and other possible confounding, non-biochemical factors (model 4). Model 5 showed that male sex, waist, systolic BP, cholesterol and current smoking, in addition to SAF and age, were independently associated with mortality. Similarly, univariate regression analyses revealed SAF to be strongly associated with both incident type 2 diabetes and incident CVD separately (Table 4). In addition to SAF and age, the strongest predictors of incident type 2 diabetes were fasting glucose, HbA_1c_, triacylglycerol, BMI, waist circumference, BP and the presence of the metabolic syndrome. The strongest univariate predictors for CVD were—again in addition to SAF and age—waist circumference, BMI, glucose, HbA_1c_, BP, eGFR and presence of the metabolic syndrome. SAF remained significantly associated with type 2 diabetes and incident CVD in the first four multivariate models. Also in these multivariate models, the presence of the metabolic syndrome, fasting glucose and HbA_1c_ levels was strongly associated with incident type 2 diabetes and moderately associated with incident CVD. In the final model (model 5), SAF still was significant, and age, glucose, waist circumference, male sex and triacylglycerol were the strongest factors associated with incident type 2 diabetes, and age, waist circumference, systolic BP and current smoking were the strongest factors associated with incident CVD.

**Table 4:** Univariate and multivariate logistic regression analyses for the separate primary outcomes (incident type 2 diabetes, CVD or death) at a median of 4 year follow-up

Analysis	New T2DM		New CVD		Death	
	OR (95% CI)	*p* value	OR (95% CI)	*p* value	OR (95% CI)	*p* value
Univariate analysis^a^						
SAF (AU)	2.74 (2.44, 3.07)	1.0 × 10^−68^	3.25 (2.93, 3.60)	2.5 × 10^−111^	5.10 (4.56, 5.70)	4.1 × 10^−181^
Age (years)	1.05 (1.05, 1.06)	1.5 × 10^−91^	1.07 (1.06, 1.07)	3.1 × 10^−184^	1.10 (1.09, 1.10)	3.4 × 10^−239^
Male sex (y/n)	1.45 (1.28, 1.64)	2.5 × 10^−9^	1.51 (1.35, 1.69)	4.3 × 10^−13^	1.64 (1.44, 1.87)	8.6 × 10^−14^
BMI (kg/m^2^)	1.16 (1.14, 1.17)	1.1 × 10^−174^	1.06 (1.05, 1.07)	3.3 × 10^−22^	1.05 (1.03, 1.06)	1.6 × 10^−11^
Waist (cm)	1.07 (1.06, 1.07)	9.2 × 10^−195^	1.03 (1.03, 1.04)	1.4 × 10^−52^	1.03 (1.03, 1.04)	4.4 × 10^−37^
Glucose (mmol/l)	12.5 (11.3, 13.9)	<1.0 × 10^−350^	1.82 (1.64, 2.02)	8.2 × 10^−30^	2.20 (1.95, 2.47)	1.6 × 10^−39^
HbA_1c_ (mmol/mol)	1.45 (1.43, 1.48)	<1.0 × 10^−350^	1.15 (1.13, 1.17)	1.5 × 10^−60^	1.17 (1.15, 1.19)	6.6 × 10^−57^
SBP (mmHg)	1.03 (1.03, 1.04)	6.3 × 10^−74^	1.03 (1.03, 1.03)	6.9 × 10^−73^	1.03 (1.03, 1.04)	1.1 × 10^−71^
DBP (mmHg)	1.04 (1.04, 1.05)	2.7 × 10^−43^	1.04 (1.03, 1.04)	1.3 × 10^−41^	1.03 (1.03, 1.04)	1.4 × 10^−23^
Heart rate (bpm)	1.01 (1.01, 1.02)	7.1 × 10^−5^	1.00 (1.00, 1.01)	0.944	1.00 (0.99, 1.00)	0.883
Cholesterol (mmol/l)	1.20 (1.14, 1.28)	5.7 × 10^−10^	1.40 (1.33, 1.48)	5.9 × 10^−37^	1.33 (1.25, 1.41)	2.9 × 10^−19^
Triacylglycerol (mmol/l)	1.44 (1.39, 1.50)	1.1 × 10^−72^	1.20 (1.15, 1.26)	1.4 × 10^−17^	1.18 (1.12, 1.24)	1.9 × 10^−10^
eGFR (ml/min)	0.98 (0.98, 0.98)	1.5 × 10^−24^	0.97 (0.96, 0.97)	1.2 × 10^−69^	0.96 (0.95, 0.96)	6.8 × 10^−91^
Former smoker (y/n)	1.44 (1.27, 1.63)	1.1 × 10^−8^	1.52 (1.36, 1.70)	6.6 × 10^−13^	1.53 (1.34, 1.75)	3.9 × 10^−10^
Current smoker (y/n)	1.11 (0.96, 1.28)	0.172	1.34 (1.18, 1.52)	7.0 × 10^−6^	1.45 (1.25, 1.67)	8.4 × 10^−7^
Coffee (cups/day)	1.08 (1.06, 1.11)	1.8 × 10^−9^	1.09 (1.07, 1.12)	7.6 × 10^−13^	1.10 (1.07, 1.13)	1.0 × 10^−10^
Metabolic syndrome (y/n)	8.9 (7.9, 10.1)	6.6 × 10^−262^	2.14 (1.88, 2.43)	1.4 × 10^−30^	2.24 (1.93, 2.60)	3.8 × 10^−26^
Multivariate model 1^a^						
SAF (AU)	1.64 (1.42, 1.90)	3.1 × 10^−11^	1.62 (1.41, 1.84)	1.5 × 10^−12^	2.37 (2.06, 2.73)	1.7 × 10^−33^
Age (years)	1.04 (1.04, 1.05)	3.5 × 10^−44^	1.06 (1.05, 1.06)	1.2 × 10^−102^	1.08 (1.07, 1.08)	2.4 × 10^−127^
Multivariate model 2^b^						
SAF (AU)	1.43 (1.22, 1.66)	4.0 × 10^−6^	1.58 (1.38, 1.80)	3.1 × 10^−11^	2.32 (2.01, 2.67)	8.8 × 10^−31^
Age (years)	1.03 (1.03, 1.04)	3.5 × 10^−26^	1.06 (1.05, 1.06)	4.2 × 10^−96^	1.08 (1.07, 1.08)	3.1 × 10^−122^
Metabolic syndrome (y/n)	7.3 (6.4, 8.3)	4.3 × 10^−208^	1.54 (1.35, 1.76)	1.6 × 10^−10^	1.44 (1.23, 1.68)	4.0 × 10^−6^
Multivariate model 3a^c^						
SAF (AU)	1.40 (1.20, 1.65)	3.0 × 10^−5^	1.60 (1.40, 1.83)	6.0 × 10^−12^	2.34 (2.03, 2.70)	1.9 × 10^−31^
Age (years)	1.02 (1.01, 1.02)	1.0 × 10^−6^	1.06 (1.05, 1.06)	2.4 × 10^−93^	1.08 (1.07, 1.08)	9.8 × 10^−116^
Glucose (mmol/l)	11.2 (10.0, 12.5)	<1.0 × 10^−350^	1.20 (1.08, 1.34)	0.001	1.29 (1.14, 1.46)	7.2 × 10^−5^
Multivariate model 3b^c^						
SAF (AU)	1.41 (1.21, 1.65)	8.0 × 10^−6^	1.58 (1.38, 1.81)	2.1 × 10^−11^	2.37 (2.06, 2.73)	1.4 × 10^−32^
Age (years)	1.01 (1.00, 1.01)	0.058	1.06 (1.05, 1.06)	1.5 × 10^−79^	1.08 (1.07, 1.08)	3.5 × 10^−107^
HbA_1c_ (mmol/mol)	1.43 (1.40, 1.46)	1.0 × 10^−265^	1.05 (1.03, 1.06)	2.0 × 10^−6^	1.02 (1.00, 1.04)	0.035
Multivariate model 4^d^						
SAF (AU)	1.32 (1.12, 1.54)	0.001	1.35 (1.18, 1.56)	2.1 × 10^−5^	1.98 (1.71, 2.30)	1.0 × 10^−19^
Age (years)	1.04 (1.03, 1.04)	1.1 × 10^−33^	1.06 (1.06, 1.07)	1.5 × 10^−103^	1.08 (1.08, 1.09)	3.6 × 10^−134^
Male sex (y/n)	0.91 (0.80, 1.04)	0.155	1.21 (1.07, 1.36)	0.002	1.32 (1.14, 1.52)	1.2 × 10^−4^
Waist (cm)	1.06 (1.06, 1.07)	1.4 × 10^−153^	1.02 (1.02, 1.02)	1.0 × 10^−14^	1.01 (1.01, 1.02)	7.3 × 10^−5^
Current smoker (y/n)	1.28 (1.10, 1.49)	0.002	1.70 (1.48, 1.94)	1.8 × 10^−14^	1.96 (1.68, 2.30)	5.0 × 10^−17^
Multivariate model 5^e^						
SAF (AU)	1.26 (1.06, 1.48)	0.008	1.33 (1.16, 1.54)	6.0 × 10^−5^	1.96 (1.69, 2.28)	6.7 × 10^−19^
Age (years)	1.02 (1.02, 1.03)	3.8 × 10^−8^	1.06 (1.05, 1.06)	3.4 × 10^−58^	1.08 (1.08, 1.09)	9.1 × 10^−84^
Male sex (y/n)	0.65 (0.57, 0.75)	6.4 × 10^−10^	1.16 (1.03, 1.31)	0.019	1.25 (1.08, 1.44)	0.003
Waist (cm)	1.03 (1.02, 1.04)	1.2 × 10^−26^	1.02 (1.01, 1.02)	2.4 × 10^−9^	1.01 (1.00, 1.02)	0.009
Glucose (mmol/l)	8.9 (8.0, 10.0)	2.0 × 10^−302^	0.96 (0.85, 1.08)	0.458	1.10 (0.96, 1.26)	0.172
SBP (mmHg)	1.00 (1.00, 1.01)	0.175	1.01 (1.01, 1.01)	2.7 × 10^−7^	1.01 (1.00, 1.01)	4.5 × 10^−4^
Cholesterol (mmol/l)	0.84 (0.78, 0.90)	9.4 × 10^−7^	1.05 (0.99, 1.11)	0.149	0.91 (0.85, 0.98)	0.014
Triacylglycerol (mmol/l)	1.24 (1.18, 1.30)	9.9 × 10^−19^	1.06 (0.99, 1.13)	0.088	1.05 (0.96, 1.14)	0.275
eGFR (ml/min)	1.00 (1.00, 1.01)	0.120	1.00 (0.99, 1.00)	0.706	1.00 (1.00, 1.01)	0.641
Current smoker (y/n)	1.22 (1.04, 1.44)	0.017	1.69 (1.47, 1.94)	7.7 × 10^−14^	1.96 (1.67, 2.30)	1.5 × 10^−16^

^a^Age-corrected

^b^Including the metabolic syndrome

^c^Including glycaemic measures

^d^Without biochemical markers

^e^With all variables

DBP, diastolic BP; SBP, systolic BP; T2DM, type 2 diabetes; y/n, yes/no

As the time of death of all participants was recorded, we were able to show the effect of SAF on time from baseline to death. As can be seen in ESM Fig. 3, the highest SAF *z* score tertile was associated with an almost twofold increased risk of mortality compared with the other tertiles.

Finally, as age is an important factor influencing SAF measurements, but also the absolute incidence of outcome events (Fig. 1), we calculated the association between age-corrected SAF score and outcome according to four clinically relevant age groups (Table 5). SAF score was significantly associated with the composite outcome and with mortality in all age groups. For incident type 2 diabetes, in participants aged ≤35 years and those between 51 and 60 years SAF was not significant. For CVD, there was no significant predictive value in the lowest age group probably because of the low number of events.

**Table 5:** Predictive value of age-corrected SAF score for the composite outcome and the three individual outcomes according to participants’ baseline age

Outcome	*n*	*n* (%) events	OR SAF *z* score	*p* value
Composite				
Age (years)				
≤35	17,412	144 (0.8)	1.86 (1.13, 3.06)	0.014
36–50	36,963	1272 (3.4)	2.02 (1.76, 2.32)	2.1 × 10^−23^
51–60	10,605	651 (6.1)	1.70 (1.40, 2.06)	6.8 × 10^−8^
≥61	7900	1064 (13.5)	1.84 (1.60, 2.12)	2.1 × 10^−17^
Type 2 diabetes				
Age (years)				
≤35	17,412	66 (0.4)	1.53 (0.71, 3.29)	0.277
36–50	36,963	489 (1.3)	2.03 (1.64, 2.52)	8.8 × 10^−11^
51–60	10,605	230 (2.2)	1.28 (0.92, 1.79)	0.140
≥61	7900	271 (3.4)	1.39 (1.07, 1.80)	0.014
CVD				
Age (years)				
≤35	17,412	53 (0.3)	1.56 (0.66, 3.71)	0.310
36–50	36,963	502 (1.4)	1.52 (1.21, 1.92)	3.1 × 10^−4^
51–60	10,605	284 (2.7)	1.62 (1.22, 2.15)	0.001
≥61	7900	419 (5.3)	1.68 (1.37, 2.06)	7.8 × 10^−7^
Mortality				
Age (years)				
≤35	17,412	25 (0.1)	3.45 (1.41, 8.44)	0.007
36–50	36,963	306 (0.8)	2.78 (2.18, 3.54)	1.8 × 10^−16^
51–60	10,605	164 (1.6)	2.47 (1.75, 3.49)	3.0 × 10^−7^
≥61	7900	433 (5.5)	2.13 (1.75, 2.60)	7.7 × 10^−14^

ORs are shown with 95% CIs

## Discussion

This prospective study within the general population demonstrates that SAF is significantly associated with new-onset type 2 diabetes, CVD and mortality during a median follow-up of 4 years. SAF predicted these combined outcomes independently of several conventional risk factors, including age, sex, waist circumference, the metabolic syndrome, smoking status, fasting glucose and/or HbA_1c_.

Both fasting glucose and HbA_1c_ were used to define type 2 diabetes at follow-up, which may have caused overestimation of their predictive values. SAF also significantly predicted mortality alone, even after correction for all relevant risk factors, such as age, sex, waist circumference and smoking. Finally, SAF was most strongly predictive in participants aged 36 and above, probably because of the low incidence of events in the lowest age group (age ≤35 years, Table 5).

The formation and accumulation of AGEs is increased in individuals with diabetes as a result of chronic hyperglycaemia and oxidative stress [8, 33]. In the present study, SAF levels were already elevated at baseline before diagnosis of type 2 diabetes, compared with people who remained normoglycaemic. Indeed, previously we demonstrated that SAF levels were strongly correlated with presence of the metabolic syndrome, a cluster of risk factors which is associated with increased risk of type 2 diabetes [21]. This association has been confirmed in the present study. However, SAF remained an independent predictor of incident type 2 diabetes, even when adjusted for presence of the metabolic syndrome at baseline. Our analyses also revealed that SAF predicted incident type 2 diabetes when adjusted for fasting glucose and HbA_1c_ levels, and it remained significantly associated even when adjusted for a large number of variables, including glycaemic measures, age, waist circumference, BP, triacylglycerol and eGFR.

Several earlier cross-sectional studies have assessed whether SAF is able to detect undiagnosed type 2 diabetes. Based on various receiver operating characteristic curves, skin fluorescence measured with the Scout DS device had higher sensitivity and specificity compared with fasting plasma glucose and HbA_1c_ in the detection of individuals with undiagnosed abnormal glucose tolerance [34]. However, these analyses were not corrected for important factors such as age, waist circumference, glucose level and smoking status. Another study compared an SAF decision model, based on age percentiles, BMI and family history, with the Finnish Diabetes Risk Score (FINDRISC) questionnaire and conventional risk markers, including fasting plasma glucose and HbA_1c_, for the detection of prevalent impaired glucose tolerance and diabetes [35]. Analyses in a subgroup of individuals, classified a priori as intermediate risk, showed that the SAF-based decision model had a higher sensitivity and specificity compared with fasting plasma glucose alone and the FINDRISC questionnaire, and had a performance equal to HbA_1c_. Finally, our group recently demonstrated in the same Lifelines cohort that measurement of SAF is of additional value to the FINDRISC for detecting current undiagnosed diabetes [36]. Reclassification analysis showed that SAF reclassified 8–15% of the total population into more accurate risk categories.

In the current study, SAF was also significantly associated with a threefold increased risk of incident CVD. This association remained significant after adjustment for age and sex, as well as the metabolic syndrome, which includes presence of elevated waist circumference, elevated BP, low HDL-cholesterol and triacylglycerol, all well-known risk factors for CVD [37, 38]. SAF remained significantly associated even after adjustment for important CVD risk factors such as actual BP levels, total cholesterol and current smoking. It has been demonstrated that tobacco smoking is a strong risk factor for a wide range of CVDs [39, 40]. Tobacco smoke is also an exogenous source of AGEs and increases oxidative stress [41–43]; both active and passive smoking significantly increase SAF [19, 20, 44]. This also suggests that the association between smoking status and risk of CVD may, in part, be explained by increased accumulation of AGEs as a result of tobacco smoking. Also, it should be noted that baseline SAF scores were the highest in individuals who developed both type 2 diabetes and CVD (Fig. 2). Although this is a small subgroup of only 55 participants, it supports the power of SAF for predicting very-high-risk individuals.

The most striking finding was that SAF was associated with a fivefold increased mortality risk in our univariate analysis. This association remained highly significant even after correcting for several confounding factors, including those described in the most extensive fifth model (Table 4). The results in Table 5 showed high ORs that are highly significant for all age groups. As this is the first study that evaluated the effect of SAF in the general non-diabetic population, we have no other study results for comparison. Although several cross-sectional studies have demonstrated the association between SAF and macro- and microvascular complications of type 2 diabetes, prospective studies regarding the predictive value of SAF are scarce and limited to selected patient populations [19, 23, 24]. SAF has been shown to be a prognostic factor for cardiac mortality in individuals with diabetes [45] and in those receiving haemodialysis [46–48]. De Vos et al have shown that SAF predicts all-cause mortality and major adverse cardiovascular events in participants with peripheral artery disease after 5 years of follow-up [25]. Moreover, in the same patient population, they found that SAF predicted lower limb amputation independently of diabetes status and disease severity after 6 years of follow-up [26]. Addition of SAF to the Fontaine classification, a method to assess severity of peripheral artery disease, improved the prediction of amputation significantly.

Both previous and present findings support the clinical utility of SAF as a first screening method for type 2 diabetes, CVD and mortality. Other risk indicators, such as presence of the metabolic syndrome, require more extensive measurements, including a fasting blood sample to measure glucose, HDL-cholesterol and triacylglycerol, but HbA_1c_ solves the need for measuring fasting glucose. The quick, non-invasive measurement of SAF may even allow use in non-medical settings or public locations such as supermarkets, pharmacies or drug stores as a first estimate of risk. The AGE reader in the present study may be used to calculate SAF percentiles using measurements in healthy participants, based on the data from Koetsier et al [20]. The present version of the device can account for both age and sex, but BMI and smoking status might also be accounted for, to produce a more balanced interpretation of the SAF value.

### Strengths and limitations

We have presented data from a prospective population-based study that included almost 73,000 participants within a broad range of age and cardiovascular risk. This is the first prospective study to examine SAF as a predictor for type 2 diabetes, CVD and mortality in the general population. Although Lifelines extensively collected information on medication use at baseline, unfortunately no data were available on the use of new medications or changes in medications, as this information was not included in the follow-up questionnaires. Medication use, in particular oral blood-glucose-lowering agents and/or insulin, can validate self-reported diagnosis of type 2 diabetes, or even ascertain the presence of diabetes when a participant does not report diabetes correctly in the questionnaire. Also, data regarding the exact time of diabetes diagnosis and CVD events were not collected. As a consequence, we were not able to perform survival analyses for both diseases. We do not have follow-up blood glucose or HbA_1c_ measurements for 16,720 participants. This may underestimate the incidence of type 2 diabetes, and could alter the effects described.

As the study has been performed in people of Western European descent, the results may not be generalisable to other populations.

Finally, future studies need to incorporate the specific cause of death in order to further refine the predictive power of SAF.

### Conclusions

This is the first prospective study in the general population to show the predictive value of SAF for incident type 2 diabetes, CVD and mortality. SAF significantly predicted the risk of these outcomes independently of several conventional risk factors. A longer follow-up of Lifelines participants will allow further validation and will expand the present findings.

## Electronic supplementary material

ESM (PDF 226 kb)

## Data Availability

The manuscript is based on data from the Lifelines Cohort Study. Lifelines adheres to standards for data availability. The data catalogue of Lifelines is publicly accessible at www.lifelines.nl. All international researchers can obtain data at the Lifelines research office (research@lifelines.nl), for which a fee is required. The Lifelines system allows access for reproducibility of the study results.

## Abbreviations

AU: Arbitrary units
CVD: Cardiovascular disease
eGFR: Estimated GFR
IQR: Interquartile range
SAF: Skin autofluorescence
