# Supplementary material for: Skin autofluorescence predicts incident type 2 diabetes, cardiovascular disease and mortality in the general population
Source: Diabetologia. 2018 Nov 21;62(2):269–80. doi: 10.1007/s00125-018-4769-x (PMC6323092; doi:10.1007/s00125-018-4769-x)

Electronic supplementary material (ESM).

ESM Methods.

About Lifelines

Lifelines is a multi-disciplinary prospective population-based cohort study examining in a unique three-generation design the health and health-related behaviours of 167,729 persons living in the North of The Netherlands. It employs a broad range of investigative procedures in assessing the biomedical, socio-demographic, behavioural, physical and psychological factors which contribute to the health and disease of the general population, with a special focus on multi-morbidity and complex genetics.

Details of the Lifelines investigations

Baseline investigation 2007-2013: two questionnaires, physical and laboratory measurements.
Interim questionnaire 1.
Interim questionnaire 2.
Second screening 2014-2018: two questionnaires, physical and laboratory measurements.

Relevant items of the baseline questionnaire

Q : Do you have diabetes mellitus ? yes / no
Q : Which type of diabetes do you have ?
    Type 1 diabetes
    Type 2 diabetes
    Other form of diabetes ?
    Do not know which type.
Q : How old were you when diabetes was diagnosed ? xx years
Q : How are you treated ?
    Diet only
    Oral blood glucose-lowering agents
    Only insulin
    Insulin and oral agents.
Q : Did you ever have a myocardial infarction ? yes / no
    If yes : how old were you when you had your first myocardial infarction ?

Q : Did you ever undergo balloon dilatation or bypass surgery ?

If yes : how old were you when you had your first procedure ?

Q : Did you ever have a stroke ?

Q : Did you ever have a disorder which was not mentioned previously ? (open text answer)

Q : A complete list of all medication, verified by the research assistant (ATC code, dose).

Relevant items of interim questionnaires 1 and 2 :

Q: Could you indicate which of the following health problems you have (had) since the last time you filled out this questionnaire ?

Diabetes

Myocardial infarction

Stroke

Clogged or blocked arteries in the legs (claudication)

Q; Did you have another severe disorder which was not mentioned in one of the earlier questions ? (Open text answer)

Q: Did you undergo a surgical procedure since the last time you filled out this questionnaire, and what was the reason for this surgery?

Balloon angioplasty for heart problems and/or bypass surgery

Other surgery ? (Open text answer)

Relevant items of second screening questionnaire

Q. Could you indicate which of the following health problems you have (had) since the last time you filled out this questionnaire ?

Diabetes yes / no

If yes, was it: Type 1 diabetes

Type 2 diabetes

Diabetes in pregnancy

Cardiovascular disease yes / no

If yes, was it: myocardial infarction

Stroke

Clogged or blocked arteries in the legs (claudication)

Data processing and validation of Lifelines measurements of skin autofluorescence

Measurements in the Lifelines study were obtained in 91443 participants using 27 calibrated AGE Readers. Recalibration by the manufacturer after repair of a device was applied 47 x during the total measurement period (2007 – 2013). These basic measurements were obtained with software version 2.1. The software obtained the spectra that were needed to calculate values of SAF and also stored these spectra in a separate file that also includes the name of the calibration file.

1. Recalculation of basic measurements

Before inclusion into the database of Lifelines, all SAF data were recalculated in cooperation with Diagnoptics Technologies B.V. with AGE Reader software version 2.3, using the stored measurement spectra per subject and the corresponding calibration files of the AGE Readers. Software version 2.3 contains a correction for skin colour if UV skin reflection ≥ 6%.

2. Validation of SAF measurements

AGE Readers and calibration cohorts were validated by normalising SAF with the expected value per participant according to Koetsier et al. [1]. Calibration cohorts with deviations >11% were excluded (2.7% of the data). Mean standard deviation of the included calibration cohorts was 6%.

Moreover, data of the triple measurements were checked for consistency: data that were based on three measurements were accepted when the standard error was < 7.2%. In that case mean SAF was applied. In other cases, the median value was applied when the standard error of the only least deviating SAF values was < 7.2%. Furthermore, SAF data were excluded for participants that reported application of sunscreen on the skin. The number of participants with SAF values that are included into the Lifelines data release of 2018 was 82904 (90.7%). Recalculation and validation were performed by R.G. totally blinded for other variables of the participants other than SAF measurement and use of skin creams.

[1] Koetsier M, Lutgers HL, de Jonge C, Links TP, Smit AJ, Graaff R (2010) Reference values of skin autofluorescence. Diabetes Technol Ther 12:399-403

ESM Table 1.

Age and skin autofluorescence for the different age groups, according to outcome

	No events			Events *		
Age range (years)	n	Age (years)	SAF (AU)	n	Age (years)	SAF (AU)
< 30	9452	24.7 ± 3.4	1.51 ± 0.27	59	25.3 ± 3.4	1.54 ± 0.29
30-39	15106	35.0 ± 2.9	1.73 ± 0.32	252	36.0 ± 2.7	1.84 ± 0.37
40-49	25567	44.7 ± 2.9	1.92 ± 0.36	956	45.3 ± 2.8	2.04 ± 0.40
50-59	11945	53.4 ± 3.0	2.10 ± 0.38	727	54.0 ± 3.2	2.20 ± 0.44
60-69	6337	63.7 ± 2.7	2.27 ± 0.42	772	64.1 ± 2.7	2.41 ± 0.48
70-79	1274	72.7 ± 2.5	2.41 ± 0.46	312	73.6 ± 2.7	2.53 ± 0.44
≥ 80	68	82.0 ± 2.4	2.51 ± 0.48	53	83.0 ± 2.6	2.68 ± 0.40

Data are mean ± SD.

* Events defined as combined outcome for incident type 2 diabetes, cardiovascular disease and mortality

ESM Fig. 1.

Flow chart of the study population.

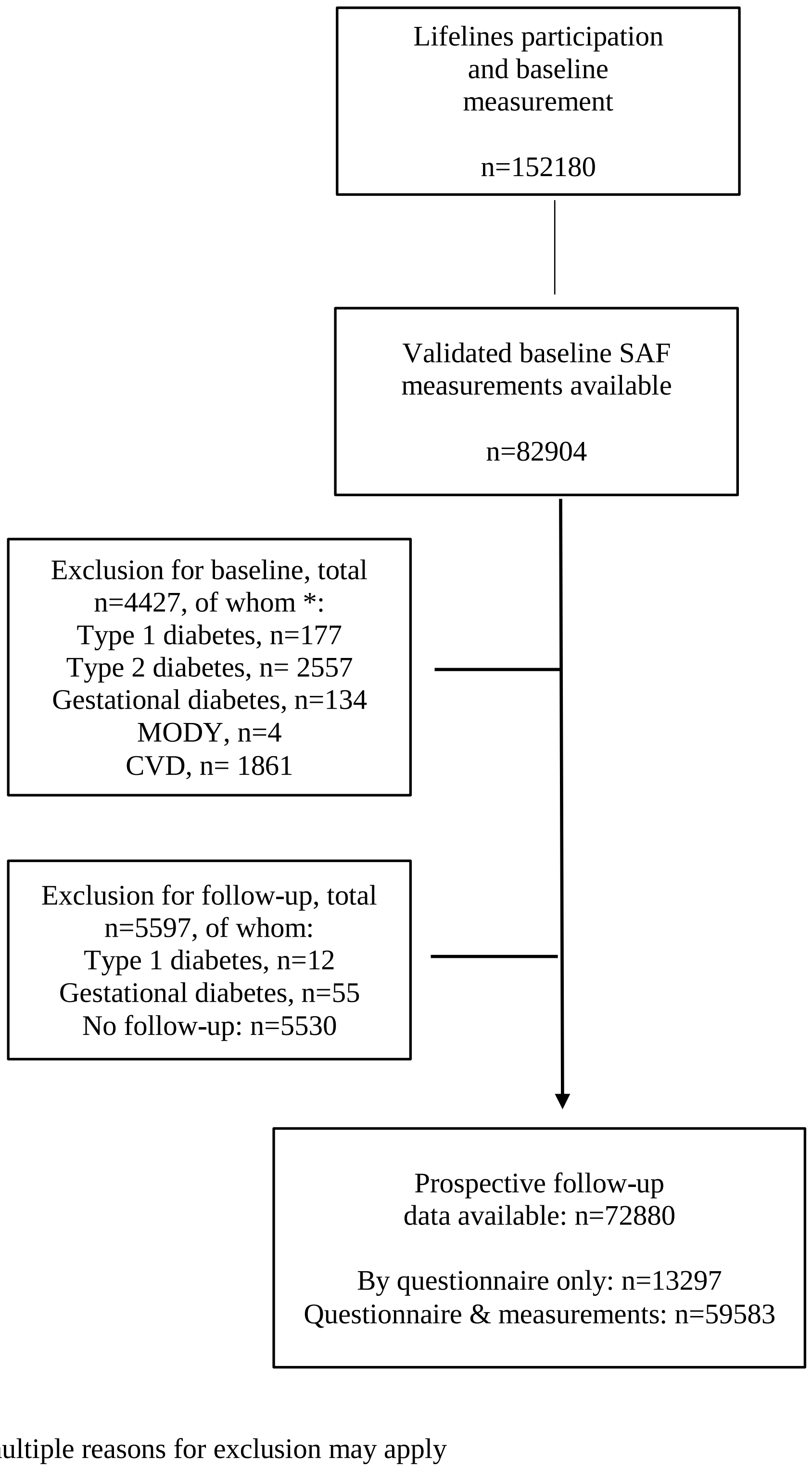

* multiple reasons for exclusion may apply

ESM Fig. 2.

Graphical representation of duration of follow-up of the participants

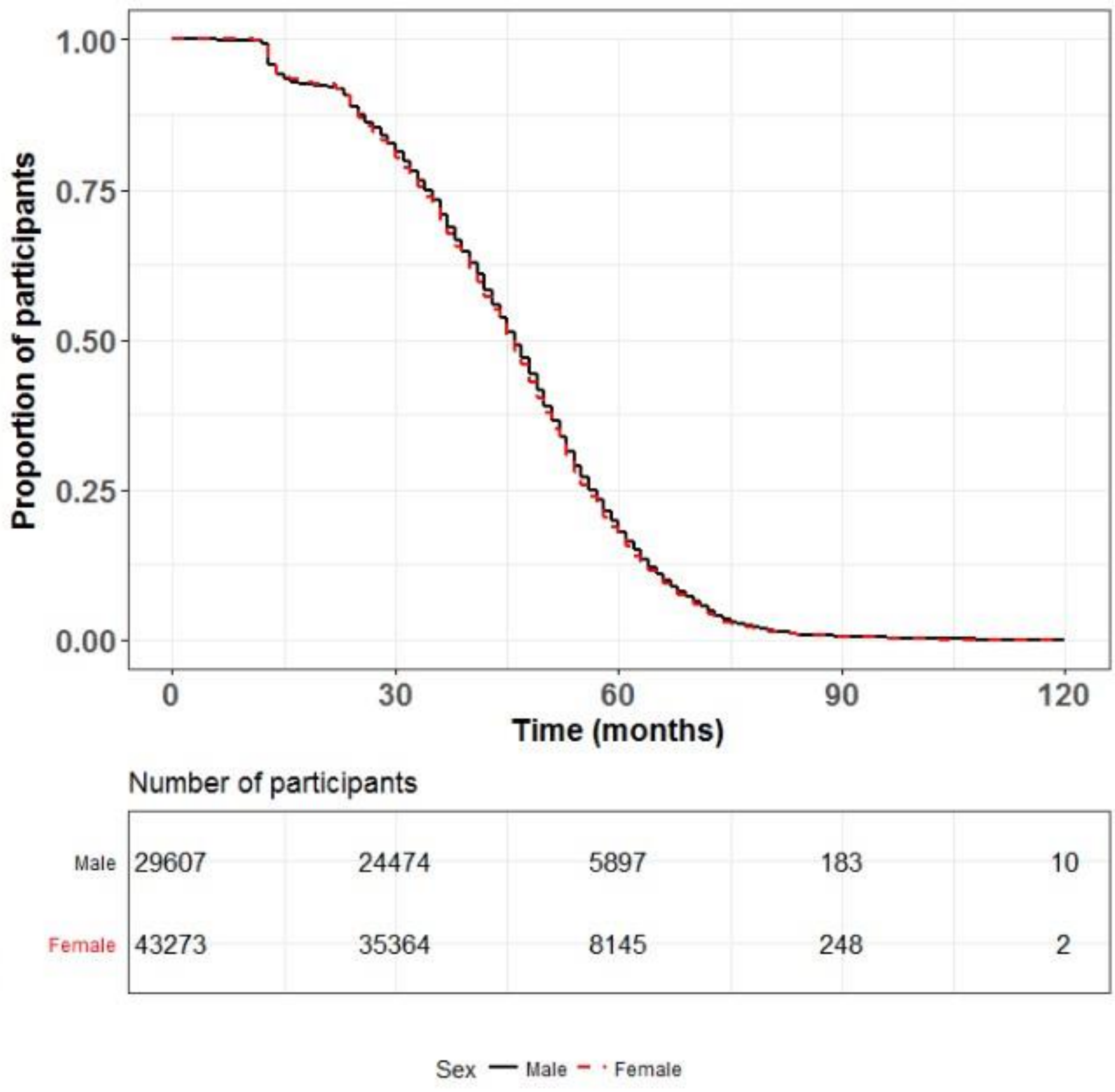

ESM Fig. 3.

Cox regression analysis depicting the effect of SAF Z-score (divided into tertiles) on mortality

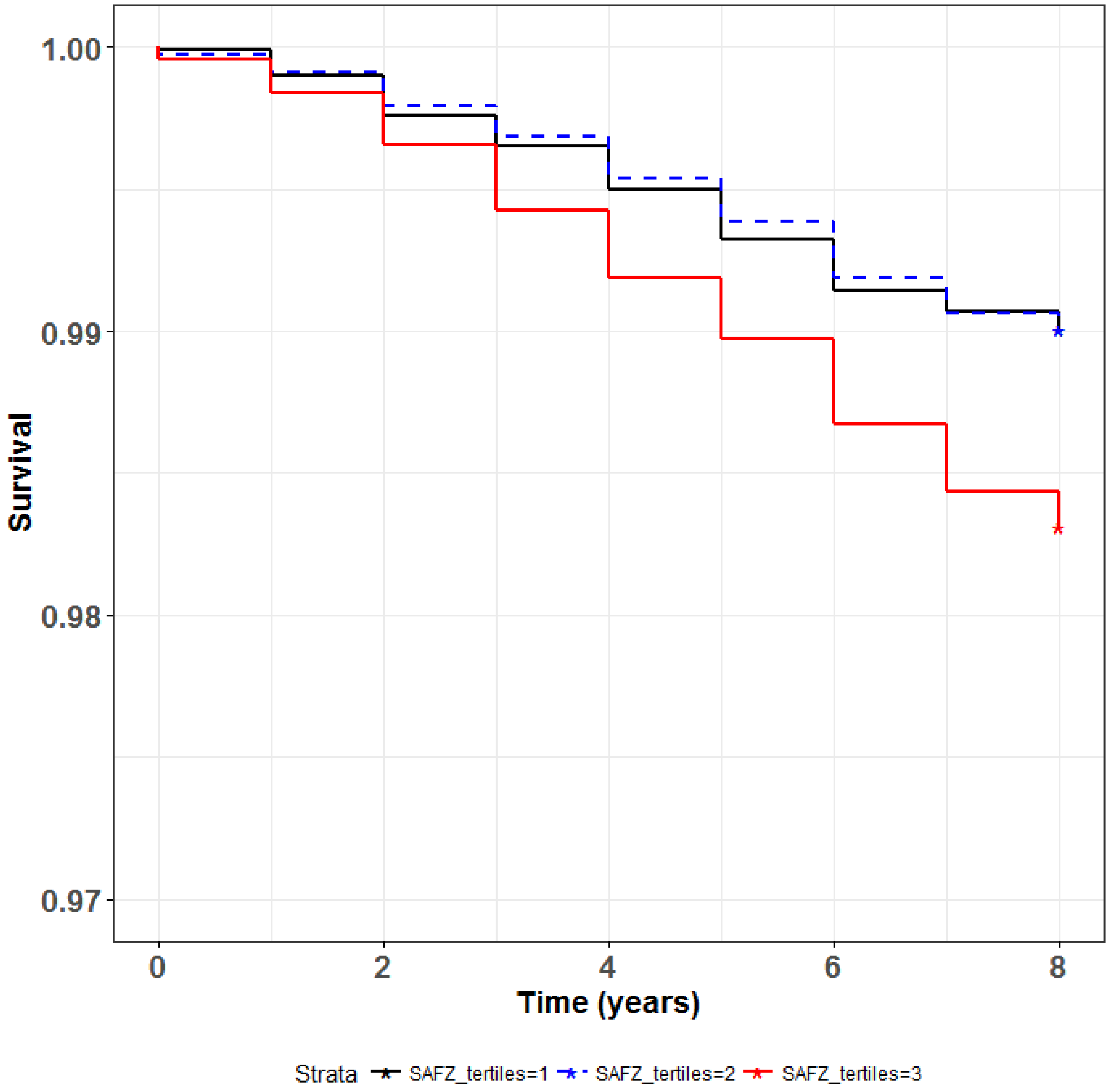

Supplement: Supplementary file 1 — (PDF 226 kb) [file 125_2018_4769_MOESM1_ESM.pdf]
